# Five Synchronous Groin Hernias (Bilateral Lateral and Femoral and Right Medial) Repaired in a Single Laparoscopic Transabdominal Preperitoneal Session: A Case Report

**DOI:** 10.7759/cureus.94137

**Published:** 2025-10-08

**Authors:** Yoichi Miyaoka, Shingo Shimada, Tomoya Saito, Ryoji Yokoyama, Akinobu Taketomi

**Affiliations:** 1 Department of General Surgery, Abashiri-Kosei General Hospital, Abashiri, JPN; 2 Department of Surgery, Otaru General Hospital, Otaru, JPN; 3 Department of Gastroenterological Surgery I, Hokkaido University Graduate School of Medicine, Sapporo, JPN

**Keywords:** ehs clinical hernia classification, femoral hernia, groin hernia, laparo-endoscopic hernia repair, myopectineal orifice, occult inguinal hernia, transabdominal preperitoneal (tapp) repair

## Abstract

Concomitant bilateral inguinal and femoral hernias are exceptionally rare, and most reports describe no more than three simultaneous groin defects. We report a 65-year-old male with a clinically diagnosed right groin bulge. During laparoscopic transabdominal preperitoneal (TAPP) exploration, the first intraperitoneal survey showed a right medial inguinal hernia (M3) and a right lateral inguinal hernia (L1), together with a left lateral inguinal hernia (L1) (European Hernia Society classification). After systematic dissection of both myopectineal orifices (MPOs), three additional occult hernias were identified, yielding five defects in total: right M3, L1, and femoral F2, plus left L1 and femoral F3. Each MPO was repaired with an anatomically contoured mid-weight polypropylene mesh (10 × 16 cm) with ≥3 cm overlap beyond all defect margins and secured using absorbable tackers. Recovery was uneventful. The patient was discharged on postoperative day three with no recurrence or complications at the latest follow-up. This case illustrates the diagnostic and therapeutic advantages of TAPP for comprehensive bilateral MPO evaluation and single-stage repair of multiple, often occult, groin hernias.

## Introduction

Groin hernias arise at weak points across the myopectineal orifice (MPO), yet true synchronous constellations that include inguinal and femoral defects on both sides are exceptional. Femoral components are often elusive on physical examination and may remain unapparent on routine preoperative imaging; when both MPOs are deliberately inspected endoscopically, previously unrecognized (occult) defects are not uncommon in laparoendoscopic series and focused reviews [1,2].

For standardized reporting, we adopt the European Hernia Society (EHS) groin hernia classification, which encodes the anatomic site as M (medial/direct), L (lateral/indirect), or F (femoral) and grades defect size as 1 (<1.5 cm), 2 (1.5-3 cm), or 3 (>3 cm or multiple) according to the intraoperative width of the hernia orifice; side and recurrence status can be appended to the code [3,4].

While addressing contralateral disease with an anterior open approach typically necessitates an additional skin incision, transabdominal preperitoneal (TAPP) permits evaluation and, when indicated, repair of both groins through the same trocar sites in a single session. Here, using the EHS framework, we report a case in which TAPP enabled single-session identification and repair of five synchronous groin hernias: right M3, L1, and F2, and left L1 and F3.

## Case presentation

**Figure 1:**
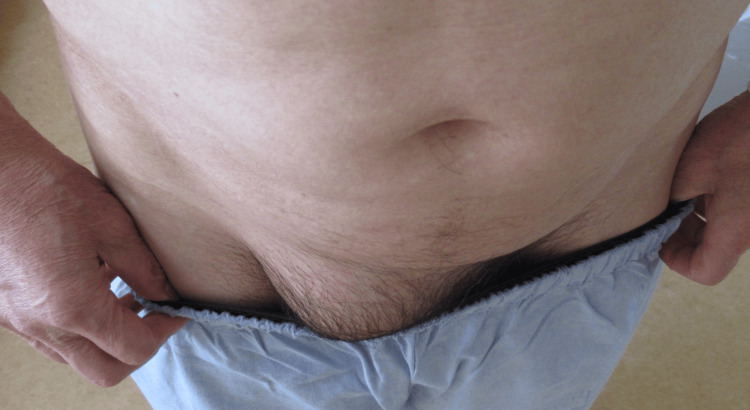
Standing examination photograph of the groin. A hen-egg-sized, soft, fully reducible bulge is present in the right inguinal region, consistent with a reducible right inguinal hernia. No swelling is observed on the left.

A 65-year-old male (165.6 cm, 57.6 kg; BMI = 20.9) presented with a four-month history of progressive right-groin swelling without bowel or urinary symptoms. Past history included ranula excision at age 25 and well-controlled hypertension on an angiotensin-II receptor blocker; he had quit smoking 20 years earlier and drank alcohol occasionally. On examination in the standing position, a hen-egg-sized, fully reducible bulge was noted on the right with no left-sided mass (Figure 1). As the clinical findings were definitive, preoperative ultrasonography and an abdominal computed tomography scan were omitted at our institution, where imaging is not routinely obtained when the diagnosis is clinically unequivocal.

**Figure 2:**
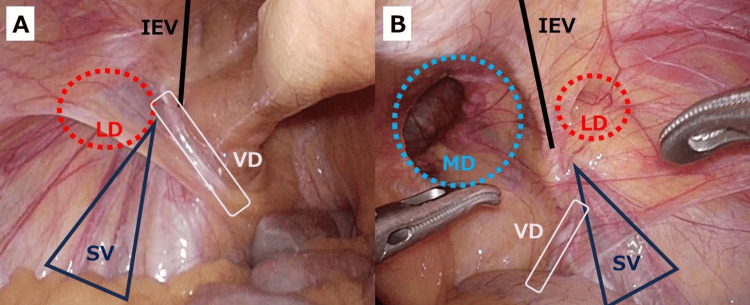
Laparoscopic view before peritoneal incision (both groins). (A) Left side: lateral (indirect) defect (LD) visible; inferior epigastric vessels (IEV) demarcate medial–lateral boundaries. Spermatic vessels (SV) and vas deferens (VD) were identified. (B) Right side: medial (direct) defect (MD) and a small lateral (indirect) defect (LD) were seen; IEV, SV, and VD labeled. IEV, inferior epigastric vessels; SV, spermatic vessels; VD, vas deferens; LD, lateral (indirect) defect; MD, medial (direct) defect.

Given the absence of prior lower abdominal surgery, a posterior laparoendoscopic approach was selected and performed. After induction of anesthesia, a urinary catheter was placed for bladder decompression. The procedure was carried out using a 12-mm umbilical camera port and two 5-mm working ports. The initial intraperitoneal survey before peritoneal incision demonstrated a right medial (M3) and right lateral (L1) defect together with a left lateral (L1) defect (Figure 2).

**Figure 3:**
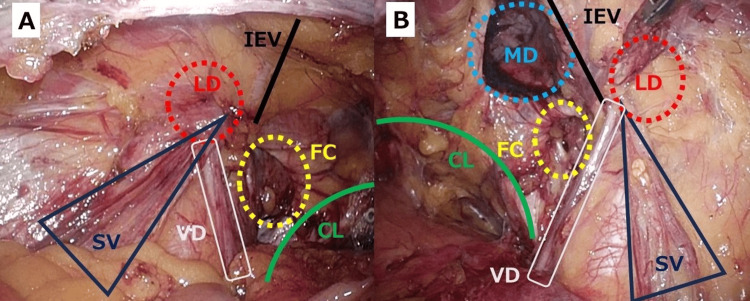
After peritoneal incision and complete MPO dissection (both groins). (A) Left side: previously noted LD; additional femoral canal (FC) defect consistent with F2 identified. Cooper’s (pectineal) ligament (CL) was visible; SV and VD were preserved. (B) Right side: MD and LD visualized; additional FC defect (F3) identified adjacent to CL. IEV, inferior epigastric vessels; SV, spermatic vessels; VD, vas deferens; LD, lateral (indirect) defect; MD, medial (direct) defect; FC, femoral canal; CL, Cooper’s (pectineal) ligament.

Following peritoneal incision and wide preperitoneal dissection of both MPOs, two additional occult femoral hernias were identified, a right F2 (1-2 cm) and a left F3 (>3 cm), yielding a final EHS classification of right M3 + L1 + F2 and left L1 + F3 (Figure 3).

All sacs were reduced, and anatomically contoured mid-weight macroporous polypropylene meshes (10 × 16 cm) were placed bilaterally to blanket each MPO with ≥3 cm overlap in all directions and fixed with absorbable tackers. The peritoneal defect was closed laparoscopically with a continuous absorbable suture at the end of the procedure. The patient ambulated on postoperative day (POD) one, resumed oral intake without difficulty, and was discharged on POD three. At two months of follow-up, he remains asymptomatic with no clinical evidence of recurrence or complications; he was instructed to seek prompt evaluation should new groin discomfort, swelling, or pain occur. No routine return visit has been scheduled, and follow-up will be arranged as clinically indicated.

## Discussion

This case adds to the sparse literature on complex multi-defect groin hernias by documenting five synchronous defects managed in a single TAPP operation. Prior case-based literature has described configurations with up to six coexisting groin hernias and underscored that missed defects can underlie persistent pain despite apparently successful repair [5,6].

A key implication is the importance of structured bilateral MPO exploration during posterior laparoendoscopic repair. When the contralateral side is inspected endoscopically, occult pathology is found at non-trivial rates: contralateral defects were identified in approximately one in five patients undergoing posterior repair initially deemed unilateral, with similar incidences reported specifically during TAPP [7,8]. A contemporary systematic review concluded that contralateral occult hernias are frequently encountered and that selective exploration and repair can be safe, while stressing individualization to patient factors and intraoperative findings [9].

Beyond inguinal defects, femoral components are regularly unmasked during laparoendoscopic repair. This is clinically meaningful because femoral hernias carry a disproportionately high risk of incarceration and strangulation, and elective repair at the index operation may avert emergency presentations and bowel resection [1,10,11]. Technique also matters: our recent case report highlighted that a final low-pressure (~8 mmHg) inspection at the end of TAPP can unmask a contralateral occult defect not visible earlier, reinforcing the value of deliberate, methodical viewing before closure [12].

From a technical standpoint, pan-MPO coverage with generous overlap remains central. International and society guidance recommends meshes of at least 10 × 15 cm for laparoendoscopic groin repair, larger for big or multiple defects, to blanket direct, indirect, and femoral spaces and thereby reduce recurrence [13]. Practical technique articles similarly advocate macroporous polypropylene meshes of roughly 12-15 × 15-17 cm with margins extending several centimeters beyond all defect edges [14]. In the present operation, 10 × 16 cm contoured mid-weight meshes were placed bilaterally with ≥3 cm overlap, consistent with these principles.

With respect to patient-centered outcomes, laparoendoscopic techniques are associated with lower rates of chronic postoperative groin pain compared with open repair, while recurrence appears broadly comparable in randomized evidence syntheses [15].

Not all patients are optimal candidates for a posterior approach. After radical prostatectomy, preperitoneal scarring and distorted planes can make TAPP/totally extraperitoneal repair (TEP) technically demanding; registry-level analyses show differing complication profiles between laparoendoscopic and open repair without clear overall superiority, supporting an approach tailored to surgeon expertise and patient factors [16]. Likewise, multiple previous lower abdominal operations remain a relative contraindication, especially for TEP, given potential preperitoneal adhesions; meta-analytic and consensus sources suggest feasibility in experienced hands but emphasize careful selection and readiness to convert or to choose an anterior open repair when posterior planes are unfavorable [13,17,18].

## Conclusions

Bilateral MPO exploration during TAPP enables single-session detection and repair of multiple, often occult, femoral defects. In this patient, five synchronous hernias were safely corrected using pan-MPO mesh coverage with adequate overlap, with an uneventful recovery. As a practice point, it is recommended to inspect both sides deliberately (including a low-pressure final look) and blanket the entire MPO.
